# Healthcare Management and Health Economics

**DOI:** 10.3390/healthcare10101879

**Published:** 2022-09-27

**Authors:** Steffen Flessa, Manuela De Allegri

**Affiliations:** 1 Department of General Business Administration and Healthcare Management, University of Greifswald, Friedrich-Loeffler-Str. 70, 17487 Greifswald, Germany; 2 Heidelberg Institute of Global Health, University Hospital and Faculty of Medicine, Heidelberg University, Im Neuenheimer Feld 130.3, 69120 Heidelberg, Germany

The Universal Declaration of Human Rights stipulates that, “recognition of the inherent dignity and of the equal and inalienable rights of all members of the human family is the foundation of freedom, justice and peace in the world” (Preamble). Consequently, equality “in dignity and rights” (Art. 1) represents the highest duty of all state authorities, and dignity is the guiding principle of all policy-making expressed in most countries’ constitutions (e.g., Art. 7, Federal Constitution of the Swiss Confederation; Art. 1, Basic Law for the Federal Republic of Germany) [1]. However, this guiding principle is very abstract. Hence, measuring the achievement of this target requires operationalisation so that this principle can be translated into specific, measurable and realistic objectives.

The Universal Declaration of Human Rights, as well as most constitutions, regard freedom, equity and solidarity as basic values exemplifying the abstract term dignity, but even these values are difficult to define and the measurement of their achievement requires operationalisation as objectives. These objectives must be inferable from the basic values; thus, they must be much more precise. Effectiveness and quality, affordability, sustainability, and participation are such objectives that can be measured and that can serve as a foundation for policy decisions [2]. Figure 1 demonstrates the system of principle value, basic values and objectives.

Human dignity is infringed upon by poor health and diseases. Thus, the provision of preventive, curative and palliative healthcare services as well as health promotion are grounded into our fundamental rights and values. The basic values underlying all policy- and decision-making in the healthcare sector are freedom, equity and solidarity, which are operationalised as effectiveness/quality, affordability, sustainability and participation. It is obvious that only effective healthcare systems and healthcare services of good quality will have an impact on the health of people. At the same time, these services must be accessible (spatial and financial) [3] so that universal health coverage (UHC) can be achieved [4]. Furthermore, healthcare services must give all stakeholders a right to participate in processes affecting them so that basic services are provided irrespective of individual characteristics, such as sex, location, age, income, etc. Finally, intergenerational justice calls for the consideration of the utility of future generations calling for the sustainability of services, i.e., future patients must find at least the same quantity and quality of services as the current generation [5].

Additionally, what does all of this have to do with health economics and healthcare management? Healthcare services are provided under the condition of limited resources, e.g., budgets, personnel and materials are scarce and require rational decisions to make the best use of them [6]. In order to protect the dignity of citizens, politicians, healthcare providers and all concerned decision-makers, we must find ways to avoid the waste of scarce resources and use them efficiently while balancing competing demands for healthcare.

Economics is the art of efficiency, i.e., it seeks ways to use scarce resources in the best way. Efficiency can be expressed as the quotient between outcomes and resources consumed to produce these outcomes. Economic systems are described, explained, analysed and designed so that this quotient is maximised. This can be achieved either by maximising the outcomes with given resources or minimising the resources consumed to produce a given outcome. The opposite of efficiency is the waste of resources, which is seen as unethical as any wasted resource cannot be used to produce meaningful outcomes for individuals and society.

$$E=\frac{Outcomes}{Resources}\to Max!$$

In order to analyse the efficiency and propose an efficient system, decision-makers must know the outcomes which are produced and the resources consumed to produce them, i.e., decisions must be based on economic evidence. The value of the latter can be easily expressed in costs as the financial value of the resources, whereas the outcomes are frequently multidimensional and cannot be standardised easily.

Health economics is also the art of designing and running an efficient healthcare system. The outcome of a healthcare system is—in principle—health which can be expressed by different indicators, such as (healthy) life years, quality of life or individual satisfaction, where the denominator is the cost. Consequently, any analysis of the efficiency of a healthcare system will start with an analysis of its costs. In other words: what resources are consumed to produce the healthcare services and the resulting population health and what is the financial value of the resources consumed to produce these services?

An improvement of the numerator in the equation, outcomes, can be achieved in three ways: First, we can increase the healthcare resources. Secondly, we can improve the technical efficiency, i.e., organise the production of healthcare services more efficiently so that no resources are wasted in healthcare institutions. Thirdly, we can improve the allocative efficiency, i.e., design healthcare systems in a way that resources are utilised where they produce the best results.

In summary, we can state that health economics provides health policy-makers, managers and other decision-makers with the instruments to make the best out of the given resources in order to achieve effective, accessible, participatory and sustainable healthcare systems and services. In this way, health economics is an instrument to fight for the basic principle of dignity of every human being. Healthcare management can be perceived as an essential element of health economics with a stronger, practical focus on the design and leadership of organisations in the healthcare system, i.e., on the technical efficiency. Although general health economics focusses on the (allocative) efficiency of the entire healthcare system, healthcare management provides more practical analyses and instruments for achieving efficiency in healthcare providers, prevention programs, health districts, insurances or other organisations.

In this Special Issue, “Healthcare Management and Health Economics” of the journal *Healthcare*, we invite submissions of papers which demonstrate the relevance and potential of health economics and healthcare management for health policy-making and public health in low-, middle- and high-income countries. We invite contributions that can range from descriptive to prescriptive scientific papers, as long as all demonstrate that health economics and healthcare management have a lot to offer for health-policy makers, managers and other decision-makers in the healthcare sector; by avoiding the waste of resources and making the best out of scarce inputs, efficiency enables the achievement of fundamental human rights and the protection of human dignity.

## Figures and Tables

**Figure 1:**
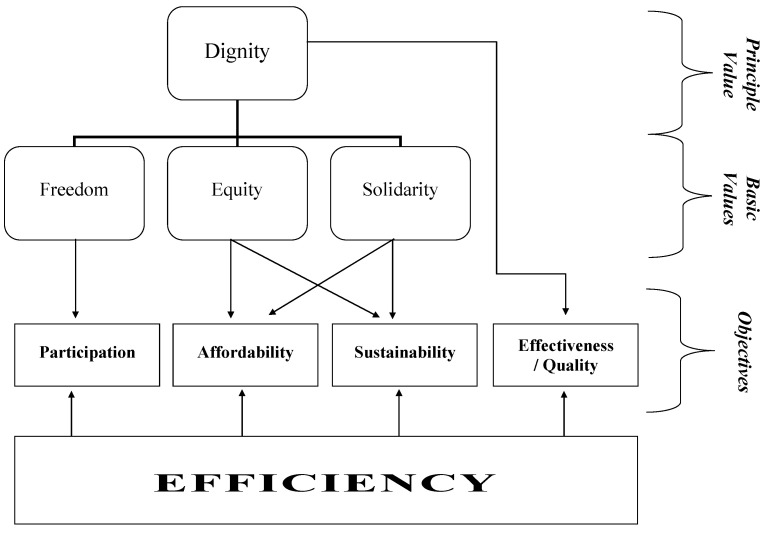
Values and objectives. Source: own, based on [2].

## Data Availability

Not applicable.

## References

[1] Walt G. (2001). Health Policy: An Introduction to Process and Power.

[2] Fleßa S., Greiner W. (2020). Grundlagen der Gesundheitsökonomie: Eine Einführung in das Wirtschaftliche Denken im Gesundheitswesen.

[3] Meade M., Emch M. (2010). Medical Geography.

[4] Evans D.B., Hsu J., Boerma T. (2013). Universal health coverage and universal access. Bull. World Health Organ..

[5] Lennox L., Maher L., Reed J. (2018). Navigating the sustainability landscape: A systematic review of sustainability approaches in healthcare. Implement. Sci..

[6] Zweifel P., Breyer F., Kifmann M. (2009). Health Economics.

